# Exposure to infection when accessing groceries reveals racial and socioeconomic inequities in navigating the pandemic

**DOI:** 10.1038/s41598-023-28194-y

**Published:** 2023-02-11

**Authors:** Daniel T. O’Brien, Alina Ristea, Sarina Dass

**Affiliations:** 1 School of Public Policy and Urban Affairs, Northeastern University, 1135 Tremont St., Boston, MA 02120 USA; 2 Boston Area Research Initiative, Northeastern & Harvard Universities, Boston, MA USA; 3 University College London, London, England

**Keywords:** Environmental social sciences, Risk factors

## Abstract

Disasters often create inequitable consequences along racial and socioeconomic lines, but a pandemic is distinctive in that communities must navigate the ongoing hazards of infection exposure. We examine this for accessing essential needs, specifically groceries. We propose three strategies for mitigating risk when accessing groceries: visit grocery stores less often; prioritize generalist grocery stores; seek out stores whose clientele have lower infection rates. The study uses a unique combination of data to examine racial and socioeconomic inequities in the ability to employ these strategies in the census block groups of greater Boston, MA in April 2020, including cellphone-generated GPS records to observe store visits, a resident survey, localized infection rates, and demographic and infrastructural characteristics. We also present an original quantification of the amount of infection risk exposure when visiting grocery stores using visits, volume of visitors at each store, and infection rates of those visitors’ communities. Each of the three strategies for mitigating exposure were employed in Boston, though differentially by community. Communities with more Black and Latinx residents and lower income made relatively more grocery store visits. This was best explained by differential use of grocery delivery services. Exposure and exposure per visit were higher in communities with more Black and Latinx residents and higher infection rates even when accounting for strategies that diminish exposure. The findings highlight two forms of inequities: using wealth to transfer risk to others through grocery deliveries; and behavioral segregation by race that makes it difficult for marginalized communities to avoid hazards.

## Introduction

The COVID-19 pandemic has had a monumental impact on society, but far from being a “great equalizer,” it has taken an outsized toll on racially and socioeconomically marginalized communities in the United States. Not only have Black, Latinx, and disadvantaged populations suffered from higher infection and mortality rates^1–3^, they have also seen disparate levels of economic and social disruption^4–6^. This is nothing new. Scholars of environmental justice (EJ) have repeatedly observed that disasters and other environmental hazards hold inequitable consequences for marginalized groups^7–12^. The protracted nature of the pandemic makes it distinct, however. Most disasters, including hurricanes and earthquakes, last as little as a few hours and at most a few days. As a result, EJ typically focuses on pre-disaster preparation for hazards^13,14^ and the ability to access remedies post-disaster^15–17^. In contrast, the COVID-19 pandemic posed a widespread threat for at least two years, depending on the definition, creating an additional consideration: how do people navigate ongoing hazards—in this case, the persistent threat of infection—to mitigate the risks that they generate? Here too there is ample opportunity for inequities to arise between communities owing to their ability to adopt strategies that can mitigate exposure to infection. This paper examines this possibility for accessing groceries, which is an ideal test case in that it is an essential need that requires visits to crowded indoor spaces that expose people to substantial infection risk^18^.

Many of the disparate outcomes arising from disasters are *inequities*—although all members of society are subjected to the same event, pre-existing conditions, including access to resources and amenities, education levels, and geography, lead populations with different racial and socioeconomic backgrounds to experience markedly different consequences. Most often, those who have been historically disadvantaged or marginalized suffer more. These inequities come in two forms. First, more affluent populations are better able to position themselves to be insulated from environmental hazards and often, by extension, relegate others to more vulnerable contexts. Seminal work on EJ has demonstrated, for instance, how waste disposal sites and other facilities that generate pollutants are often alongside poor and minority communities^7,19^, creating elevated risk in general and especially large concerns if there is a spill or other breakdown in infrastructure. Others have shown how redlining across the United States has confined Black communities to the warmest neighborhoods, increasing their exposure to health-threatening summertime heat^20^. Second, disadvantaged populations are less capable of accessing resources and remedies post-disaster, thus hindering local recovery and setting them further behind their affluent counterparts. For instance, vulnerable populations in Puerto Rico saw greater delays in the return of the electric grid^17^. Hurricanes Katrina and Harvey in the Gulf Coast embodied each of these forms of disaster-related inequities: more affluent communities were better protected from flooding^13,14^; and their residents were more able to afford the technology and materials to clear contaminated debris after the disaster^15^. Further, Allen^16^ describes how the formal management of recovery is such that the deficits that poor residents experience in early stages of the process make it harder for them to succeed thereafter.

The COVID-19 pandemic bears the hallmark of pre- and post-disaster inequities. Minority and disadvantaged populations entered the pandemic with higher rates of at-risk conditions (e.g., obesity, hypertension, diabetes, asthma)^21–24^ and lower access to health care^25^, elevating vulnerability to the disease^3^. They also were disproportionately represented in client-facing jobs that either were forced to continue working in-person despite the added risk (e.g., nurses) or saw severe cuts in employment (e.g., restaurant workers)^4^. There are also indications that communities of color have struggled with economic recovery relative to their White counterparts and will continue to do so^5,6,26^. But between pre- and post-disaster, communities have been forced to navigate an ongoing pandemic for over a year, circumstances that have incentivized multiple strategies for mitigating the risk of infection exposure. Crucially, these strategies might be more available to some populations than others, creating inequitable consequences.

It is well-established that disadvantaged communities were less capable of maintaining social distancing guidelines during the early months of the pandemic^27,28^. Much of this is attributed to residents being over-represented in client-facing professions, but it is also possible that inequities in accessing essential needs further contributed to these disparities. We propose three main strategies that people might adopt to lower their exposure risk when accessing essential needs. These could be generalized to any amenity or resource that is part of routine life, though here we illustrate with the case of purchasing groceries. This is an ideal case because it combines one of the utmost needs, which cannot be voluntarily delayed for very long, with crowded indoor spaces that have been demonstrated to pose a high risk of infection exposure and even measurably contribute to community-level infection rates^18,29^. Each of these three strategies also presents potential inequities rooted in pre-pandemic conditions.

1. *Visit the grocery store less often.* There are two tactics for lowering trips to the grocery store without actually changing where one shops, each of which are more accessible to the more affluent. First, households can “pantry stock,” making fewer, larger purchases, though this requires the liquid capital to do so. Alternatively, they can order grocery delivery from Instacart or a similar service. This adds an expensive premium to the food and will likely only be available to those with high incomes^30^. Grocery delivery services are particularly pernicious in terms of inequities as higher income households are transferring risk of exposure to infection to those who are making the deliveries, a population that is more likely to be low income.
2. *Prioritize generalist grocery stores.* Households could shift their grocery store visits to major supermarkets that tend to carry most needed items, foregoing alternative shops for specialty items or “bargain hunting”. This would have the dual benefit of diminishing total visits and potentially visiting larger stores, which might in turn have fewer visitors per square foot. In theory, this option should be available to everyone provided they have access to a major supermarket, though this is not a guarantee for disadvantaged and marginalized communities, who often suffer from food deserts^31,32^. Further, even where marginalized communities have access to groceries, they tend to have limited access to major chain stores^33^—the very types of stores that we suggest would be the optimal destination if trying to limit exposure during a pandemic.
3. *Visit grocery stores whose clientele have lower infection rates.* This strategy relies on knowledge (or assumptions) about the distribution of infections across a region and the grocery stores the different communities tend to frequent. First, it is unclear that most individuals would be aware of infection rates, however, and even less likely they know which communities frequent which grocery stores. As such, this strategy may be implemented by only a small slice of the population. This limitation in turn highlights the way that exposure at amenities may have been inequitably embedded in pre-pandemic patterns. There is mounting evidence that cities are not only residentially segregated but also behaviorally segregated, with communities of different demographic backgrounds largely moving in parallel through different parts of the metropolitan area^34,35^, often frequenting different amenities even when in the same neighborhood^36,37^. This reinforces isolation between racial and socioeconomic groups^38,39^, in turn concentrating the experience of challenging and deleterious experiences for racial minorities and disadvantaged individuals^40^. For instance, Levy et al.^41^ found that disadvantaged communities whose mobility patterns primarily connect them to other disadvantaged communities had the highest rates of COVID-19 over the first year of the pandemic. Visits to major amenities, such as grocery stores, may be one mechanism by which these patterns of reinforced disadvantage occur. It is likely that those living in the most impacted communities tend to visit grocery stores (and other amenities) where risk of infection is high. Further, it would be difficult for them to alter these routines to visit grocery stores where risk is substantially lower because those locations are outside of their daily routines. Because infection rates in the early pandemic were highly correlated with race and socioeconomic status, these visitation patterns would have created a feedback loop by which communities of color that were not especially hard-hit were still exposed to the challenges of other disadvantaged communities because they frequented the same places.

The current study uses a unique combination of complementary data sets to examine the strategies that the communities of greater Boston adopted when accessing groceries during April 2020, the period that saw the most acute impacts on public health and, accordingly, elicited the strongest behavioral responses for mitigating risk^42^. In particular, we assess racial and socioeconomic inequities in the ability to employ each of the three proposed strategies for limiting the risk created by accessing groceries. First, we use anonymized cellphone-generated GPS records to observe how patterns of grocery store visitation shifted with the onset of the pandemic. Second, a survey of Bostonians enables us to look more closely at certain details that are not apparent from the mobility data, including the use of grocery delivery services. Third, we use localized infection cases provided by local public health authorities to present an original calculation of infection risk exposure through grocery visits that incorporates: the volume of visitors to each grocery store; the infection rates of the communities of these visitors; and grocery store size, as a proxy for the ability to socially distance ^43–45^. This goes beyond previous efforts that have used only a subset of these components. We also use additional data sources to describe demographic differences in behaviors and exposure and to identify conditions or mechanisms that might be responsible for such relationships, including access to stores, perceptions of risk, and local infection levels. As such, we determine the extent to which disparities are a product of inequities in the ability to pursue strategies for mitigating risk.

## Results

### Shifts in visiting grocery stores

**Figure 1:**
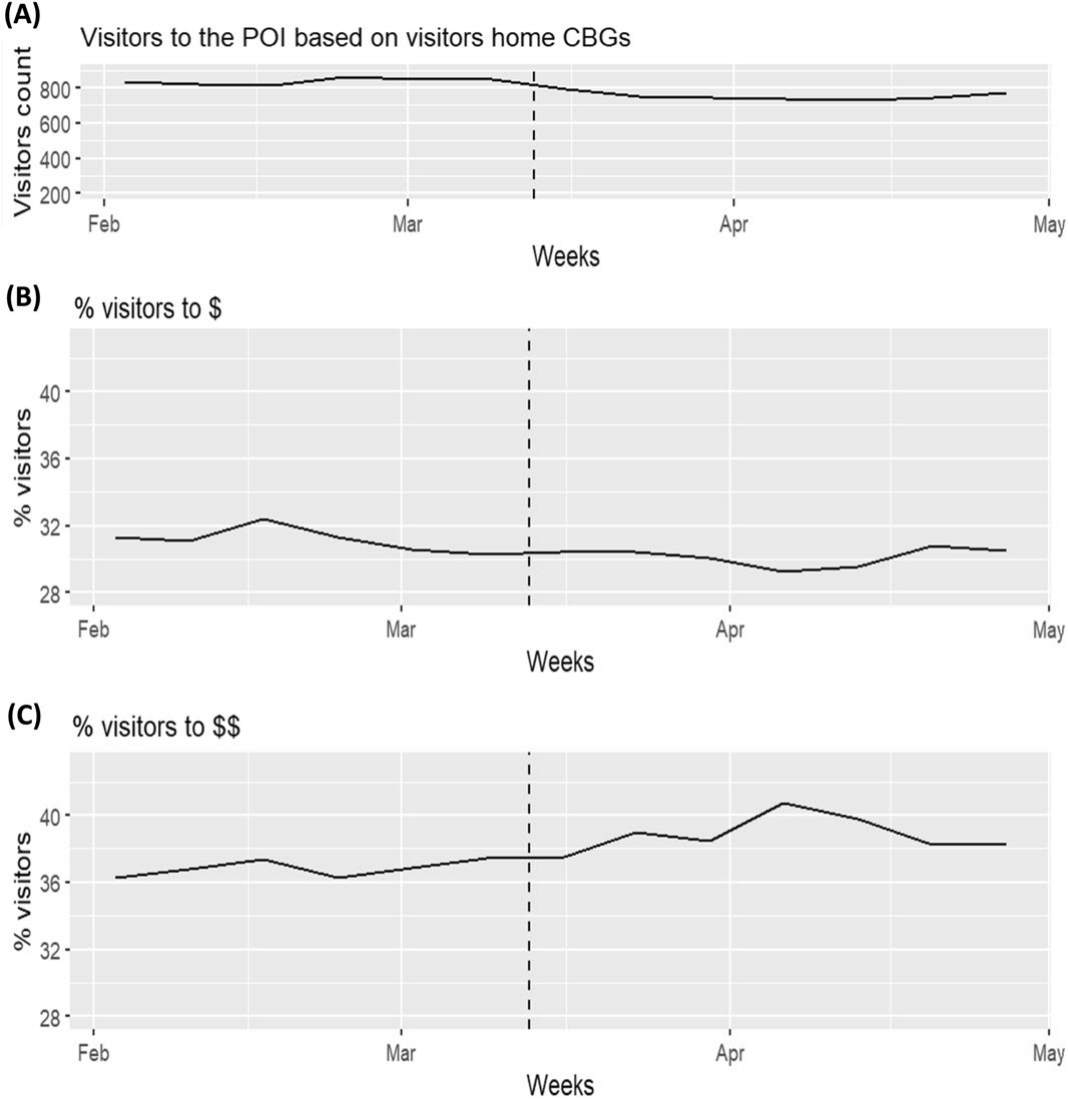
(**A**) There were moderate drops in total visits to grocery stores at the beginning of the pandemic, with a partial bounceback, accompanied by (**B**) a drop in the percentage of visits to one-dollar sign stores and (**C**) an increase in the percentage of two-dollar sign stores.

Boston’s neighborhoods lessened their visits to grocery stores between February and April 2020 by 39% (15.6 fewer visits per 1,000 residents; see Fig. 1A). On the surface, these changes varied modestly by the ethnic composition of communities. Those with greater than 30% Black residents or 30% Latinx residents each averaged a 36% drop (18.9 and 20.0 fewer trips per 1,000 residents, respectively). Majority White communities averaged 38% fewer visits (14.5 fewer trips per 1,000 residents). See Table 1 for full results.

**Table 1:** Descriptive statistics for total visits to grocery stores and distribution across types of grocery stores in February and April 2020, split by ethnic composition of neighborhood.

	Black ≥ 30%			Latinx > = 30%		
	Mean (SD) Feb	Mean (SD) Apr	%Δ	Mean (SD) Feb	Mean (SD) Apr	%Δ
Total visits	56.4 (28.2)	36.3 (20.1)	− 35.8%	57.5 (29.1)	37.0 (22.1)	− 35.6%
% $ trips	25.3 (13.8)	21.5(16.7)	− 3.8%	37.7 (15.6)	34.0 (19.5)	− 3.7%
% $$ trips	54.1 (16.5)	63.0 (20.1)	8.9%	39.1 (17.8)	46.7 (22.2)	7.6%
N	207	268				

	White ≥ 50%			Mixed population		
	Mean (SD) Feb	Mean (SD) Apr	%Δ	Mean (SD) Apr	Mean (SD) Feb	%Δ
Total Visits	38.1 (24.19)	23.6 (17.05)	− 38.2%	43.4 (24.62)	26.3 (18.64)	− 39.4%
% $ Trips	26.0 (18.3)	23.8 (20.3)	− 2.2%	31.2 (15.9)	28.0 (23.2)	− 2.8%
% $$ Trips	51.1 (20.3)	59.1 (24.6)	8.0%	46.9 (18.8)	51.1 (24.9)	4.2%
N	1936	143				

2,453 CBGs in metro Boston with population > 200 residents. Categorization by ethnicity is imperfect, as 56 CBGs had both >  = 30% Black and Latinx residents (13% of all CBGs with >  = 30% of either group) and 45 CBGs with majority White population had >  = 30% Black or Latinx residents (11% of all CBGs with >  = 30% of either group). Mixed population is all CBGs not reaching any of the three thresholds.

Drops in visits were concentrated at some types of grocery stores more than others. We use cost (or “dollar sign”) classifications to differentiate between types of grocery stores, noting that midrange (i.e., “two-dollar sign”) grocery stores tend to be larger, permitting social distancing, and have broad, reliable inventory, enabling visitors to limit their total number of trips (see Supplementary Information (SI) for validation). Indeed, trips to one- (i.e., “budget”) and three-dollar sign (i.e., “high-end”) stores dropped by half (48% and 50%, respectively), whereas trips to two-dollar sign stores dropped by under a third (29%). As illustrated in Fig. 1B–C, there was a consequent rise in the proportion of trips to two dollar-sign stores (Δ = 8.2%) at the expense of other types of stores. This again was largely consistent across communities as categorized by ethnicity, though communities with large Latinx populations and Mixed Population (i.e., not reaching any of the stated thresholds) saw less prioritization of two-dollar sign stores (Δ = 7.6% and 4.2%, respectively).

### Who changed their visitation patterns?

Various community features predicted the extent to which communities lessened their visits to grocery stores (see Methods for more detail on specification and Table 2 for main results; full models control for population size and pre-pandemic versions of the outcome measure, thereby enabling an interpretation of how other variables affected shifts in grocery store visitation; all parameters reported in SI). Communities with a higher proportion of Black and Latinx residents and lower median income had more visits than expected (% Black: B = 0.05, *p* < 0.001); % Latinx: B = 0.05, *p* < 0.001; income: B =  − 0.12, *p* < 0.001). These relationships were independent of the number of grocery stores within a 15-min drive, which was a non-significant predictor.

**Table 2:** Parameter estimates from regression models testing the relationship between race and socioeconomic status and patterns of grocery store visits in April 2020 relative to the same measures in February 2020.

	Total visits during COVID^a^	% Visits to $$ stores	% Visits to $ stores
% Black	0.045*** (0.004)	2.73*** (0.59)	− 1.02* (0.47)
% Asian	− 0.007 (0.004)	− 0.45 (0.54)	1.05* (0.42)
% Latinx	0.050*** (0.005)	− 1.16 (0.65)	2.55*** (0.51)
Med. HH income	− 0.12*** (0.005)	1.49* (0.64)	− 2.23*** (0.51)
R^2^	0.50	0.17	0.20

Sample of 2,363 CBGs in metro Boston, MA with population > 250 residents and values on all variables. All variables scaled before analysis, meaning parameters are an estimate of the increase or decrease with each change in 1 SD in the predictor variable. All models also controlled for the same measure in Feb. 2020, total population, population density, total stores or $$ or $ stores within a 15-min drive of the centroid of the CBG (as appropriate per model), % < 18 yrs., % > 65 yrs., and % commuting by car. See SI for all parameters.

^a^Counts model only from a zero-inflated Poisson model. Zero-inflation model reported in SI. *R*^*2*^ reported as McFadden’s pseudo-*R*^*2*^*.*

Communities also differed in their tendency to shift toward two-dollar-sign stores. Those with a higher percentage of Black residents especially prioritized two-dollar-sign stores (relative to predominantly White communities; B = 2.73, *p* < 0.001), whereas communities with a higher proportion of Latinx residents marginally did the reverse (B =  − 1.16, *p* < 0.10). Communities with higher median incomes also prioritized two-dollar-sign stores (B = 1.49, *p* < 0.05). These results were independent of the number of two-dollar-sign stores within a 15-min drive, which was non-significant.

To better understand what trips to two-dollar-sign stores replaced (or were replaced by) across communities, we replicated the analysis for one-dollar-sign stores. Here we see that communities with more Latinx residents and, to a lesser extent, those with more Asian residents, made a greater proportion of their visits than expected to one-dollar-sign stores (% Latinx: B = 2.55, *p* < 0.001; % Asian: B = 1.05, *p* < 0.05), whereas communities with more Black residents and higher median incomes moved away from such stores (% Black: B =  − 1.02, *p* < 0.05; med. income: B =  − 2.23, *p* < 0.001). These results were independent of the number of one-dollar-sign stores nearby, which only moderately predicted more reliance on such stores (B = 1.24, *p* < 0.10).

It is possible that cross-community variation in grocery store visitation patterns was driven by experiences or attitudes pertaining to COVID. To test for this, we first added local infection rates in April to the model, finding that communities with more infections per capita made fewer grocery trips (B =  − 0.005, *p* < 0.01). We then incorporated a survey-based measure of perceived risk from infection (though this required us to limit to Boston rather than the metropolitan region; see Methods). Communities whose residents perceived higher risk of infection made fewer trips than expected (B =  − 0.10, *p* < 0.01), holding all other variables constant. Each of these relationships did little to alter the previous findings (though there were some moderate differences between the Boston-only sample and the full region; see SI for full details). Perceived risk and local infections, however, did not predict shifts to or from one- or two-dollar sign stores.

### The impact of grocery delivery

**Figure 2:**
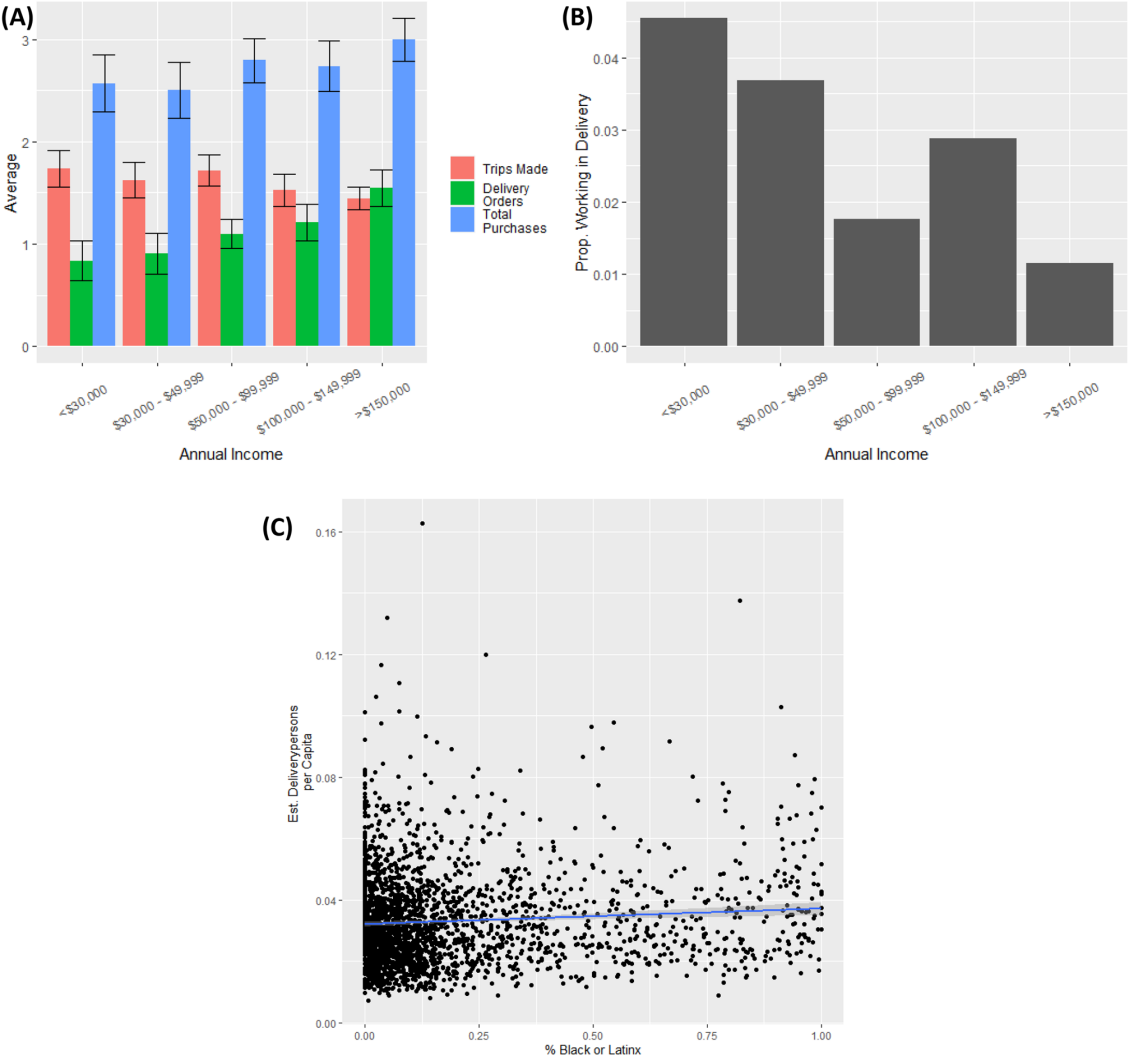
Disparities in grocery store visits were driven in part by delivery services. (**A**) More affluent residents reported making fewer trips than others but substantially more delivery orders, resulting in more total purchases. Meanwhile, (**B**) lower income survey respondents were more likely to report working in delivery services and (**C**) SafeGraph estimated a higher number of delivery workers per capita in census block groups with more Black and Latinx Residents.

A survey of Boston residents during the early months of the pandemic offers an additional set of insights into grocery store visits, particularly the extent to which households offset these with deliveries. The survey replicated the findings from the SafeGraph data as Black and Latinx respondents made visits to the grocery store more often (*means* = 2.07 and 1.71 days per week, respectively) than White and Asian respondents (*means* = 1.51 and 1.48 days per week, respectively; ANOVA: *F* = 7.23, *p* < 0.001). Likewise, those in lower income brackets made trips on more days per week (B =  − 0.09, *p* < 0.001; see Fig. 2A).

White respondents ordered grocery deliveries more often than other respondents, though the difference was only significant relative to Black respondents (*means* = 1.20 vs. 0.90 days). More affluent respondents had deliveries on more days (B = 0.16, *p* < 0.001). Interestingly, when we sum grocery store visits and deliveries, differences in total purchases across ethnicities were no longer present (*F* = 1.74, *p* = *ns*; though the specific comparison between White and Black respondents was still significant, *means* = 2.72 vs. 2.92, *p* < 0.05) and more affluent respondents made *more* purchases (B = 0.06, *p* < 0.05; see Fig. 2A). This suggests that the use of grocery delivery explained much of the racial and socioeconomic differences in grocery store visits and that little could be attributed to “pantry stocking.”

The flipside of grocery delivery services is the question of who is delivering those groceries. Though numbers were small, more Black and Latinx respondents indicated they did delivery work (4.1% and 3.8%, respectively) than White and Asian respondents (2.2% and 1.5%, respectively). Likewise, delivery reached a high of 4.5% for those making less than $30,000/yr. and fell to 1.2% for those making more than $150,000 (see Fig. 2B; trend in a logistic regression: B =  − 0.22, O.R. = 0.80, *p* < 0.05). This confirms that a crucial part of the strategy of White, more affluent households for mitigating risk exposure was through grocery deliveries made predominantly by lower income, often minority, individuals.

We complemented this analysis by examining the proportion of residents in each CBG inferred by SafeGraph is working in delivery (see Methods for more; full model presented in SI). In keeping with the survey results, we find that communities with more Black (B = 0.90, *p* < 0.001) and Latinx (B = 1.27, *p* < 0.001) residents and lower median incomes (B =  − 1.37, *p* < 0.001; see Fig. 2C) had more resident delivery workers. This proportion predicted more total visits from a community (B = 0.001, *p* < 0.001) when added to the previous models, though did little to account for ethnic and socioeconomic differences.

### Differences in exposure from grocery visits

**Figure 3:**
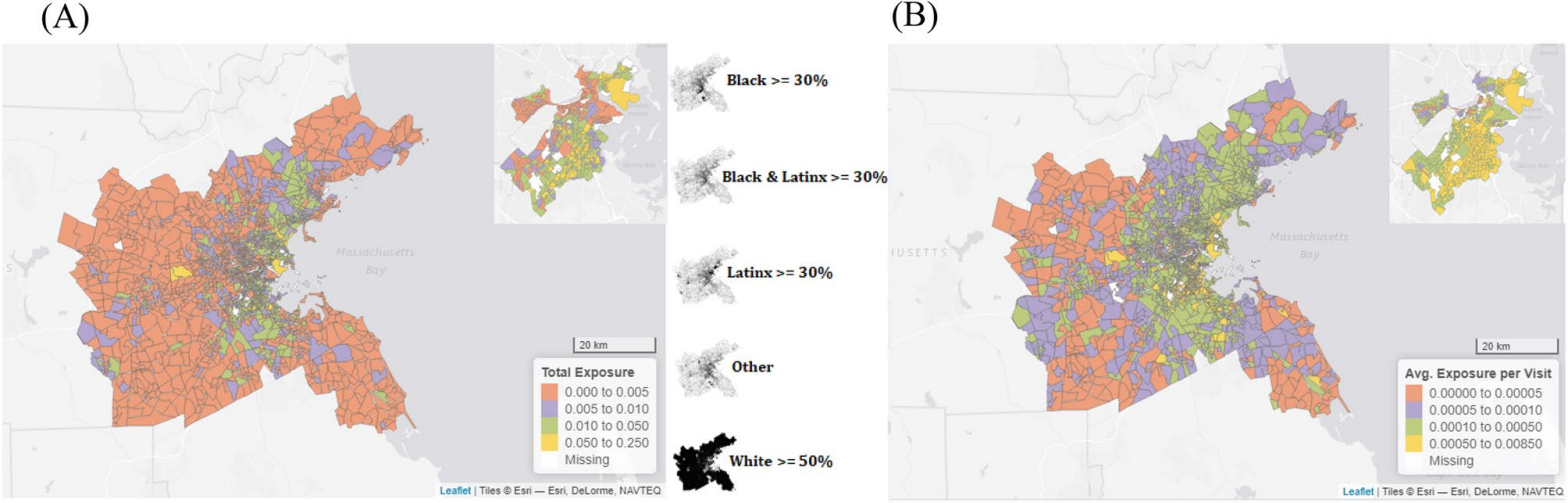
Communities of color experienced (**A**) more exposure to infection through visits to grocery stores and (**B**) more exposure per grocery store visit, especially in Boston proper (see insets; made with leaflet package in R^52^).

We estimated the exposure risk of each grocery trip from the volume of visitors, neighborhood infection rates, and store square footage and then summed exposure across all trips made by the residents in each neighborhood (see Methods for more detail, Table 3 for main results, and Fig. 3 for illustration; full model reported in SI). Communities with a higher proportion of Black, Asian, and Latinx residents and lower median income saw more total exposure from grocery store visits (% Black: B = 0.33, *p* < 0.001; % Asian: B = 0.15, *p* < 0.001; % Latinx: B = 0.27, *p* < 0.001; med. income: B =  − 0.28, *p* < 0.001). Many of these effects were expected given that they were the same community features that were associated with more visits to grocery stores. Communities with a higher local infection rate in April also saw more total exposure, which is notable because those communities made fewer trips (B = 0.10, *p* < 0.001).

**Table 3:** Parameter estimates from regression models testing the relationship between race and socioeconomic status and exposure to infection from grocery store visits in April 2020, taking into account strategies for diminishing exposure.

	Total exposure through visits	Avg. exposure per visit	Avg. exposure per visit
% visits to $$ stores	–	–	− 0.018*** (0.004)
% Black	0.33*** (0.006)	0.27*** (0.004)	0.30*** (0.004)
% Asian	0.15** (0.007)	0.15*** (0.004)	0.14*** (0.004)
% Latinx	0.27*** (0.007)	0.21*** (0.005)	0.19*** (0.005)
Med. HH income	− 0.28*** (0.01)	− 0.06*** (0.006)	− 0.04*** (0.006)
Infection rates (April 2020)	0.10*** (0.006)	0.13*** (0.003)	0.13*** (0.003)
Pseudo-R^2a^	.39	.38	.40

Sample of 2,153 CBGs in metro Boston, MA with population > 250 residents, at least one recorded grocery store visit in April 2020, and values on all other variables. The outcome variables were modeled as a Poisson distribution. All predictor variables scaled before analysis, meaning parameters are an estimate of the likelihood of increase or decrease with each change in 1 SD in the predictor variable. All models also controlled for the same measure in Feb. 2020, total population, population density, total stores or $$ or $ stores within a 15-min drive of the centroid of the CBG (as appropriate per model), % < 18 yrs., % > 65 yrs., and % commuting by car. See SI for all parameters and Methods for variable details.

^a^Reported as McFadden’s pseudo-*R*^*2*^*.*

When examining the average exposure per visit, which accounts for the volume of visits, the results were very similar. Communities with more Black, Asian and Latinx residents experienced more exposure on the average trip (% Black: B = 0.27, *p* < 0.001; % Asian: B = 0.15, *p* < 0.001; % Latinx: B = 0.21, *p* < 0.001), as did communities with higher local infection rates (B = 0.13, *p* < 0.001). Communities with lower median income also saw more exposure on average, though the effect size was substantially diminished (B =  − 0.06, *p* < 0.001).

We reran this last model twice more, once each with the proportion of trips to one-dollar-sign stores and two-dollar-sign stores, to determine the influence of this strategy on exposure per trip across communities. The proportion of two-dollar-sign-stores visited predicted a lower level of exposure per trip (B =  − 0.18, *p* < 0.001). The proportion of one-dollar-sign stores predicted the opposite (B = 0.13, *p* < 0.001). Because these effects were mirror images, we only report the results of the former in Table 2. Notably, however, these effects do not explain any of the demographic effects from the original models, though they did diminish the effect of Latinx population by 10%. Additionally, as a robustness check for the range of ways that visiting two-dollar-sign stores might impact exposure, we confirmed that doing so predicted fewer total visits (added to the count model reported in Table 2: B =  − 0.10, *p* < 0.001). This indicates that, as hypothesized, prioritizing two-dollar-sign stores lowered exposure both by diminishing the number of grocery store visits and the exposure during each of those visits.

## Discussion

Communities of different racial and socioeconomic compositions experienced markedly different levels of exposure to infection when accessing groceries stores at the beginning of the pandemic. These results complement our current understanding that inequities regularly arise from pre-disaster positioning and post-disaster opportunities^7–17^ by demonstrating similar disparities in the ability to navigate an ongoing disaster, in this case a protracted pandemic. As with previous disasters, marginalized communities, including those with more Black and Latinx residents and lower incomes, were hit harder by the pandemic^1–6^, and the current analysis found that greater exposure to infection risk when accessing basic amenities may have contributed to these disparities. Importantly, we see clear strategies for diminishing exposure, but inequities in how they might be pursued across communities, including: the use of wealth to transfer risk to others through grocery deliveries; strategy in prioritizing major supermarkets; and behavioral segregation, especially by race and ethnicity. Importantly, these results appear to be independent of well-established inequities in access to grocery stores^31–33^.

Higher income communities and those with more White residents made relatively fewer visits to grocery stores in April 2020. It has already been shown that disadvantaged communities were less able to remain at home during the early months of the pandemic^27,28^, though the public narrative has often focused on disparities in the ability to work remotely. This adds nuance to current knowledge by highlighting the concurrent role of accessing essential needs. The importance of being able to limit visits was especially apparent considering that income was no longer associated with exposure when we controlled for the quantity of visits made to stores.

We tested two hypotheses for how wealth might enable fewer visits. The first hypothesis was “pantry loading,” or making larger purchases that limit the need for follow-up visits. The second was ordering from grocery delivery services, which charge an expensive premium. A survey of residents found that not only were higher-income and White respondents more likely to use delivery services, which was also true pre-pandemic^30^, but that when the frequency of deliveries was summed with grocery store trips, racial disparities in number of purchases were nearly eliminated and more affluent respondents made *more* purchases. This indicates differences in trips were driven by the use of grocery delivery services and not by pantry loading. Further, both the survey and SafeGraph data indicate that those delivering the groceries were predominantly lower income and people of color. As such, those with more resources were not just paying to limit their own exposure—they were paying those with fewer resources to shoulder that burden on their behalf.

We observed a strategic shift toward two-dollar-sign stores, which are predominantly larger grocery stores that can be considered “one-stop” shops. We saw evidence that this can lower infection exposure both by limiting the need for supplementary trips and by offering more space to socially distance (contingent on the number of other patrons; as captured in average exposure per visit). This strategy was not evenly distributed across communities, however. Higher-income communities and those with more Black residents embraced this strategy the most. Communities with more Latinx residents did the opposite, emphasizing one-dollar sign stores, which are often corner markets. Though there is existing evidence that marginalized communities have less access to major chain stores^33^, this last finding appeared to be intentional and not merely a product of access to certain types of stores, as the number of stores of a given type nearby did not significantly predict these shifts. Though one might argue that smaller stores could be safer because they attract fewer customers from other neighborhoods (and therefore unknown infection likelihood), we found that focusing on two-dollar-sign stores indeed lowered average exposure per visit.

Last, we see segregation playing a strong role in driving exposure. Communities with more residents of color and more infections experienced more exposure, even when accounting for the total number of visits and the tendency to visit one- or two-dollar-sign stores. This extends previous findings that those from communities of color were more likely to visit amenities with a greater volume of visitors^43^ by taking into account the local infection rates of those visitors. Importantly, it means that communities of color were placed at greater risk for exposure when visiting grocery stores *over and above* their local infection rates and *even if* they used strategies intended to mitigate such risks. This points to the difficulty of accessing contexts different from one’s own in a city that is de facto racially and socioeconomically segregated^34,35,37^, as well as the persistent exposure to hazards for communities of color through daily mobility, independent of their local context^40^. In this case, infections were concentrated in communities of color and the consequence of segregation is that their residents had little recourse to escape the threat of exposure. This is the very definition of inequity.

There are a few limitations we must address. First, the study is of a single metropolitan region, which was necessary given our utilization of a unique combination of data sources, including surveys and infection cases at the CBG level. Nonetheless, the behavioral strategies of Bostonians may differ from other regions of the country and it would be important to replicate the findings. Second, we only analyze visits to a single type of amenity. As noted, we chose groceries as they present the unique combination of being an unavoidable necessity that is most often accessed in-person in crowded spaces where infection exposure can be high. This differs from most other amenities and thus combining it with those that might be delayed (e.g., medical treatment) or occur in less risky venues (e.g., parks and recreation) would require consideration of a distinct set of strategies and thus a separate analysis. Future research should pursue these extensions. Third, we use dollar-sign classifications (i.e., estimated relative cost) to quantify a tendency to prioritize generalist stores that tend to be larger and have full inventory. This is a rough proxy, though preferable to the alternatives. We explored the possibility of creating a category of the most common “major” grocery stores in greater Boston but found that there was no notable shift toward such stores after the onset of the pandemic (53.4% vs. 53.7% of visits in February and April, respectively). As such, this theoretically intuitive measure was failing to capture strategies in grocery access across communities and thus was irrelevant to our research questions. Fourth, we use official infection records, which are known to undercount actual case rates. That said, evidence suggests that this undercounting is amplified in low-income and majority-minority neighborhoods relative to the general population, meaning that our results probably understate inequities in exposure^46,47^. Fifth, owing to data availability, we used 2014–2018 ACS estimates for our demographic variables. It is feasible that some population shifts between then and 2020 could introduce error into our analyses. That said, the metrics we use here tend to correlate extremely highly (i.e., *r*’s > 0.9) from one ACS estimate to the next, suggesting that such error will be limited.

Additionally, although SafeGraph data are a distinctive resource for studying human mobility, they carry their own set of limitations. Because of cross-community differences in the use of cellphones, some demographic and geographic populations may be under- or overrepresented in the data. We believe we have largely controlled for such biases in our models by controlling for both total population and mobility measures from February 2020. The latter is especially important as we anticipate that these biases were consistent across time. It is of course still possible, however, that the pandemic altered the biases in some ways, lowering the efficacy of these controls. In addition, the need to suppress visitation counts from a given CBG to a given store under 4 obscures an unknown number of visits in non-systematic ways. We hope, however, that these are few enough relative to the totality of visits to have limited effect. Also, we were unable to test an additional strategy for limiting exposure, which is to make grocery store visits shorter. Analyses using more granular mobility data have suggested that people have used this tactic, especially in areas where infection rates were high^48^. Additionally, the structure of the SafeGraph data required the analysis to occur at the community level, creating potential concerns about ecological inference^49^. For example, it is not possible here to determine whether more affluent or White residents living in disadvantaged communities or communities of color are having the same experiences as their neighbors when accessing groceries. Last, because SafeGraph data aggregates visits into counts, we do not know how long individuals spent in the grocery store on each trip. For instance, it is possible that those visiting smaller stores spent less time inside, leading to a lower exposure than our estimations would assume.

In sum, we see evidence for an additional mechanism contributing to racial and socioeconomic inequities during the pandemic: the role of wealth, strategy, and segregation in determining the ability to mitigate exposure to infection risk when accessing essential amenities. The illustration here was with grocery stores, but it might feasibly be extended to other needs and institutions, including schools, parks^50^, and healthcare. This dynamic also adds to existing theory on EJ and disasters, which has focused on factors influencing pre-disaster positioning and post-disaster resources, by demonstrating the inequitable dynamics of navigating threats during an ongoing, protracted disaster (Supplementary file 1).

## Methods

The study used data from the metropolitan area including the 101 municipalities surrounding Boston, MA. Analyses were conducted at the scale of census block groups (CBGs), which are a good proxy for neighborhoods (avg. pop. approx. 1,000 people) and the most granular level of analysis for which many of the key datasets and variables were available. The analysis was limited to 2,453 CBGs with at least 250 residents, though models only included the 2,363 CBGs with values on all variables. Analyses utilizing a survey of Boston neighborhoods (see below) were limited to the 160 CBGs meeting the same criteria within the city. We compare grocery store visits in April 2020 to February 2020 as the most recent pre-pandemic period. The analytic approach was modeled on that of a related study by the authors on parks and exposure^50^.

### Data sources and measures

We used four data sources: (1) cross-community mobility records derived from cell phone records, generated by SafeGraph, a data company that aggregates anonymized location data from numerous applications in order to provide insights about physical places, via the Placekey Community; (2) population descriptors from the American Community Survey’s (ACS) 2014–2018 five-year estimates; (3) a random-sample neighborhood survey on perceptions, attitudes, and experiences during the early months of the pandemic; and (4) monthly COVID-19 case counts derived from official infection records provided by Boston Public Health Commission (BPHC). We accessed or aggregated all data at the CBG level. As noted below, in a few cases data were unavailable at this level and we imputed values from higher levels of aggregation.

### Cellphone-generated mobility records

SafeGraph is a private company that collects and aggregates mobility data from cellphones through contracts with specific mobile applications. Each mobile application obtains opt-in consent from its users to collect anonymous location data (i.e., not associated with any name or email address) with latitude and longitude recorded periodically provided one or more partner applications is open on the device. SafeGraph uses these records to (1) identify all “stay points,” or places the device visited, and (2) estimate the home CBG of the device based on its most common nighttime location. SafeGraph also curates a canonical list of “points of interest” (POIs), or amenities and destinations, including grocery stores. During the pandemic SafeGraph signed data use agreements with universities, allowing researchers to access the data freely under the SafeGraph COVID-19 Data Consortium in order to study the impacts of the pandemic through mobility data (https://www.safegraph.com/blog/safegraph-provides-cdc-fed-and-1000-organizations-with-data-to-fight-the-covid-19-crisis). Through this arrangement we had access to the Social Distancing metrics, Place Patterns, and POI data. Our process for working with these data was modeled on that reported in previous related studies on parks and exposure^50^.

We used SafeGraph’s Place Patterns dataset to measure grocery store visitation patterns across communities. SafeGraph also identifies all stay points occurring within each POI, treating them as “visits.” The Place Patterns data set presents these stay points in aggregate as a mobility matrix of the daily number of visits by the assumed residents of each CBG to each POI. To enhance privacy, SafeGraph suppresses counts of visits from a given CBG to a particular POI in a month if there was only 1 and setting any value between 2 and 4 equal to four. We accessed the subset of this matrix that quantified visits from each CBG to each grocery store in each month (we also used weekly counts for the visualization of global trends in Fig. 1). Grocery stores are pre-labelled as such in the SafeGraph data. We identified 995 grocery stores in our study area. Additionally, we use SafeGraph’s estimate of the number of devices (i.e., individuals) whose movements indicate working in delivery services, defined as having stopped for < 20 min at > 3 locations outside of their home area (note that this determination is made by SafeGraph before releasing the public data, which do not contain length-of-stay information, either for individual visits or in aggregate).

The outcome variables of interest for each CBG were calculated in February and April 2020. These measures included: *total number of visits* to grocery stores, summed across all grocery stores for each CBG; and *% of visits to categories of grocery stores* based on their “dollar sign” rating, calculated by summing all visits to grocery stores in that category and dividing by the total number of visits. The former variable was found to have a zero-inflated Poisson distribution. We also created measures of access to grocery stores from SafeGraph’s POI data set as the number of grocery stores or grocery stores of a given dollar sign rating within a 15-min drive (i.e., isochrone) from the centroid of the CBG (using the ORS Tools plug-in for QGIS).

We acknowledge that SafeGraph’s data may give a clearer view of grocery store trips in some communities than others, dependent on the extent to which residents are represented in SafeGraph’s data. In some studies this would call for controlling for the number of devices per capita identified in each community. In the current case, however, this is unnecessary because in each model we include the same measure in February 2020 as a control, thereby measuring shifts in behavior during the pandemic rather than absolute measures. This should account for any biased representation across communities as said bias should be approximately the same between February and April 2020.

### Census indicators

We drew population descriptors from the U.S. Census’ American Community Survey’s 2014–2018 estimates for all CBGs in Massachusetts using the *tidycensus* package in R^51^, which queries the U.S. Census Bureau’s public-facing data APIs. Community indicators included total population, population density, ethnic composition (i.e., proportion Asian, proportion Black, proportion Latinx, proportion White), median household income, age composition (i.e., % residents under 18 and over 65), and % commuting by car (to control for transportation access).

### Infection data

Boston Public Health Commission provided officially reported COVID-19 infection cases mapped to the tract level through a Data Use Agreement with the Boston Area Research Initiative. We aggregated these monthly for April–August 2020 to estimate the total infection risk in each tract. Beginning April 14th, 2020 the Commonwealth of Massachusetts’ Department of Health released weekly counts of new infections for all municipalities and daily counts for counties. We created daily town measures by: tabulating the weekly sum of infected cases in a county; calculating the percentage of a county’s cases attributed to each municipality; estimating the daily infected cases per municipality as the same percentage of the daily count for the county. Because neither data source for infection rates was a granular as CBGs, we imputed rates to that level, assuming that all CBGs in the same municipality shared similar infection rates, except in Boston, where we were able to impute CBG-level infection rates from census tracts, which are considerably smaller than municipalities.

### Survey data

The Boston Area Research Initiative (BARI) at Northeastern University, the Center for Survey Research (CSR) at University of Massachusetts Boston, and the Boston Public Health Commission (BPHC) conducted the Living in Boston During COVID survey in July 2020. The survey consisted of items measuring respondents’ experiences during the first months of the COVID-19 pandemic, including their ability and tendency to follow social distancing recommendations; attitudes towards regulations; and the economic and personal impacts of the pandemic. This survey was also utilized in similar ways in a previous study by the authors on parks and exposure^50^ The survey utilized a stratified random sample design that divided the city of Boston into 25 distinct neighborhoods based on social, demographic, and historical salience. Four neighborhoods with a higher proportion of Black or Latinx populations were oversampled (Hyde Park, Mattapan, Lower Roxbury, and East Boston-Eagle Hill). The survey was also administered online to members of a previously-constructed panel that had been recruited using the same 25 neighborhood stratified sample design. The final sample included 1,626 respondents who gave their informed consent and completed the survey (response rate = 26.88%). Despite the stratified sampling design, low income and non-White Bostonians were underrepresented in the final sample, for which reason we implement a post-stratification weight to better match Boston’s ethnic and socioeconomic composition for all reported statistical tests. The survey was approved by the University of Massachusetts Boston’s Institutional Review Board. The survey methodology, including stratified sampling design, quantification of post-stratification weights, and respondent demographics are reported in the SI.

We used the survey data in two ways. First, we used individual-level responses to confirm racial and socioeconomic disparities in grocery store visits, deliveries, and working for a delivery service. Respondents reported how many days on the average week in April they visited a grocery store, visited a food pantry, and ordered delivery from a grocery store. These were coded as 7 days, 5 or 6 days, 3 or 4 days, 1 or 2 days, and zero days. We recoded these as their midpoints (e.g., 5 or 6 days = 5.5). We also summed visits to grocery stores and food pantries as a single measure of trips for groceries. Respondents also reported whether their work at the time of the survey or any time since March 2020 had involved delivering food or other goods to people’s homes.

Second, we calculated tract-level measures by taking the average of a given measure for all resident respondents, weighted for non-response bias within neighborhoods. These were then imputed to the CBG level (i.e., all CBGs in the same tract had the same values). We utilized two measures from the survey. Perception of infection risk was measured with 3 items reflecting concern for oneself and family members regarding COVID-19 (e.g., “In your opinion, how much of a risk to your health and well-being is it to be within 6 feet of people in public?”; α = 0.80). High-risk behaviors were measured with 4 items reporting the frequency with which the respondent engaged in behaviors that were likely to place them at elevated risk for exposure to COVID-19 infection during in the past 7 days when completing the survey (in summer 2020). These included eating at a restaurant, bar, or club; visiting someone else’s home; attending any kind of event where more than ten people were gathered; or having people who do not live with you in your home, either to work or visit (α = 0.65). Because the survey was only of residents in Boston, excluding surrounding municipalities, it is important to note that models using these metrics were limited to those communities (*n* = 531 CBGs).

### Estimating exposure

A major part of the study was to test whether strategies of grocery store trips did or did not mitigate exposure to infection risk. Total exposure was calculated as follows (using a variant of an equation originally presented in a previous study on parks and exposure^50^):

$$Total\,Exposure\left( h \right) = \mathop \sum \limits_{g} \frac{{\mathop \sum \nolimits_{t,g} p_{t} *v_{t,g} }}{{A_{g} }}*v_{h,g}$$

where *h* is the CBG of interest, *g* is a grocery store, *t* is any of the CBGs that visited that grocery store, *v* is visits from a given CBG to a given grocery store, *p*_*t*_ is infection rate in a given CBG, and *A*_*g*_ is area in square feet of a given grocery store. In this way total exposure sums the estimated number of individuals visiting the grocery store who might be infected with COVID-19 (per the infection rate of their home neighborhood) for all visits from CBG *h* to all grocery stores, divided by the area of the grocery store as a proxy for the ability to socially distance. This follows the models of previous studies using place visitation data to estimate risk exposure^43–45^. Average exposure per visit to a grocery store was then derived from this calculation by dividing by the total number of visits made by residents of *h*, or:

$$Avg.\,Exposure\,per\,trip\left( h \right) = \frac{{\mathop \sum \nolimits_{g} \frac{{\mathop \sum \nolimits_{t,g} p_{t} *v_{t,g} }}{{A_{g} }}*v_{h,g} }}{{\mathop \sum \nolimits_{g} v_{h,g} }}$$

Both the total and average exposure metrics were very small as calculated, as a function of divergent scales of the incorporated elements (e.g., infection rates were < 0.1, and counts of visits were small relative to the divisor of square footage). They were also Poisson-distributed. To make them accessible to modeling using a logit link, we multiplied total exposure by 1,000 and average exposure by 100,000 and then rounded to the nearest whole number.

### Analysis

We used generalized linear models to assess racial and socioeconomic differences in grocery store visitation and resultant exposure and to identify conditions or mechanisms that might be responsible for such relationships, including urban form, perceptions of risk, and local infection levels. Total visits was a count variable with a long tail and a large number of zeroes in both February and April 2020. For this reason, we ran zero-inflated Poisson regressions to isolate “true zeroes,” or places that should not be expected to have any visits in any month. True zeroes are not our primary interest so we report the count model in the main text and the zero-inflation model in the SI. These models control for count of visits in February and whether there were any visits in February. Total and average exposure were Poisson-distributed without zero-inflation, requiring a standard Poisson link for models. Other outcome measures were normally distributed, justifying traditional linear models. All predictor variables were scaled before analysis, meaning parameters are an estimate of the increase or decrease with each change in 1 SD in the predictor variable.

### Statement on research ethics

All methods and protocols, including the survey and its integration with other data sets, were carried out in accordance with relevant guidelines and the expectations of partners contributing data, as affirmed by the Institutional Review Board at UMass Boston (Study ID #2020104) and accepted through an inter-university Authorization Agreement.

## Supplementary Information

Supplementary Information.

## Data Availability

The ACS indicators are publicly available through the Boston Area Research Initiative’s Boston Data Portal (BDP) on the Dataverse Network (https://dataverse.harvard.edu/dataset.xhtml?persistentId=doi:10.7910/DVN/XZXAUP). The survey data are available from the corresponding author (DTO) upon request. The SafeGraph and BPHC data were accessed through data use agreements and cannot be shared publicly. The SafeGraph data can be requested from the company through a partnership with Dewey (https://www.deweydata.io/) at the time of this writing, as data for this time period have been made accessible to academic researchers seeking to study behavior during the pandemic. The BPHC data is subject to HIPAA regulations, thus those interested would need to work with the corresponding author (DTO) to communicate with BPHC about the potential for access.
