# Supplementary material for: Exposure to infection when accessing groceries reveals racial and socioeconomic inequities in navigating the pandemic
Source: Sci Rep. 2023 Feb 11;13:2484. doi: 10.1038/s41598-023-28194-y (PMC9922100; doi:10.1038/s41598-023-28194-y)
Supplement: Supplementary file 1 — Supplementary Information. [file 41598_2023_28194_MOESM1_ESM.docx]

**Supplementary Online Materials**

This supplement contains three components: validation of the use of two-dollar-sign stores as reflective of a strategy to visit large, generalist stores (Appendix A); additional details on the survey methodology (Appendix B) and full model results (Appendix C).

*Appendix A: Validation of Two-Dollar-Sign Stores*

There were 356 two-dollar-sign stores identified by SafeGraph in the greater Boston area. These tended to be larger than other stores (average of 33,041 sq. ft. vs. 18,084 sq. ft. for stores with other classifications; *t*-test: *t* = 4.87, *p* < .001). Unpacking this, we conducted an ANOVA test across categories, findings that there were significant differences in size across dollar-sign classifications (*F*-test = 9.90, *p* < .001). Two-dollar-sign stores indeed were the largest and were significantly larger than one-dollar-sign stores and those with no classification (*p* < .01 for both comparisons using Tukey-HSD tests), but were not significantly different from three-dollar-sign stores. This latter point was not entirely surprising as the three-dollar-sign category is dominated by Whole Foods, which tends to have large stores that are well-known to be expensive. We also found that having a higher percentage of visits to two-dollar-sign stores indeed predicted fewer total visits when added to those models (B = -0.001, *p* < .001; see Appendix B for baseline models).

*Appendix C: Covid in Boston Survey Methodology*

The NSF-Beacon survey is a collaboration of the Boston Area Research Initiative (BARI) at Northeastern University, the Center for Survey Research (CSR) at University of Massachusetts Boston, and the Boston Public Health Commission (BPHC), funded by the National Science Foundation’s Human-Environment and Geographical Sciences (HEGS) program through a grant for rapid-response research (RAPID) for collecting ephemeral data during or following a crisis. The survey captures the experiences of 1370 Bostonians during the first months of the COVID-19 pandemic, including ability and tendency to follow social distancing recommendations, attitudes towards regulations, and economic and personal impacts of the pandemic. The design allows for a unique observation of neighborhood-level estimates for these factors.

**I. Sample Design and Final Sample**

The sample design for the NSF-Beacon survey was a stratified random sample that divided the city of Boston into 25 distinct neighborhoods. These neighborhoods were defined based on social, demographic, and historical salience in collaboration with members of the Mayor’s Office and other experts. They were constructed to conform to census block group boundaries, meaning that metrics associated with these geographies (for the U.S. Census Bureau and elsewhere) could be linked with the data. The Marketing Systems Group (MSG) was contracted to draw a simple random sample of residential addresses from within each neighborhood. They used the most recent United States Postal Service Computerized Delivery Sequence File (CDSF) to draw Address Based Samples (ABS) of residential addresses. Four neighborhoods with a higher proportion of Black or Latinx populations were oversampled (Hyde Park, Mattapan, Lower Roxbury, and East Boston-Eagle Hill). As shown in Table 1, there were unbalanced sample sizes and selection probabilities across neighborhoods, meaning that data analysis will need to incorporate survey weights to correct for these differences. In addition to the survey being administered to the sample obtained for the NSF-Beacon study, the survey was also administered online to participants in the previously-constructed Beacon panel, which had been recruited using the same 25 neighborhood stratified sample design.

**II. Data Collection Methodology**

Paper copies of the survey, plus instructions for completing and returning, and a $2 cash incentive were mailed to all sampled addresses. For three neighborhoods known to have higher percentages of Hispanic households, the materials mailed, including the survey instrument, were in both English and Spanish. All recipients were also given the option of completing the survey online and an associated URL. A randomly assigned half of the mailed questionnaires had instructions for the oldest adult 18+ in the household to complete the survey while the other random half had instructions for the youngest adult 18+ to complete the survey. In this manner, an attempt was made to randomize the age of the respondent within the household completing the survey. Approximately two weeks after the initial mailing of materials, a second mailing was sent to nonrespondents, though with no additional incentive.

**Table B1. NSF-Survey neighborhood sampling specifications**

| **Neighborhood** | **# of Sampled Addresses** | **Prob. of Selection** | **# of Completed Surveys** | **Response Rate^1^** |
| --- | --- | --- | --- | --- |
| Allston | 192 | 0.01702 | 51 | 28.81% |
| Back Bay | 194 | 0.01871 | 53 | 31.36 |
| Beacon Hill | 204 | 0.03593 | 53 | 30.11 |
| Brighton | 187 | 0.00839 | 58 | 31.87 |
| Central | 198 | 0.06119 | 50 | 27.78 |
| Central Northeast | 196 | 0.02839 | 58 | 33.14 |
| Central West | 200 | 0.01665 | 55 | 32.35 |
| Charlestown | 190 | 0.02286 | 62 | 34.25 |
| Dorchester Central | 189 | 0.01042 | 39 | 21.08 |
| Dorchester North | 188 | 0.02661 | 42 | 23.86 |
| Dorchester South | 191 | 0.01671 | 60 | 32.97 |
| East Boston | 189 | 0.02501 | 43 | 24.29 |
| East Boston-Eagle Hill | 355 | 0.04189 | 93 | 27.84 |
| Fenway/Kenmore | 195 | 0.01169 | 39 | 21.91 |
| Hyde Park | 364 | 0.02967 | 59 | 17.10 |
| Jamaica Plain | 188 | 0.01138 | 71 | 39.66 |
| Jamaica Plain-Mission Hill | 191 | 0.02737 | 55 | 30.73 |
| Lower Roxbury | 372 | 0.05977 | 57 | 17.59 |
| Mattapan | 362 | 0.02704 | 61 | 17.58 |
| Roslindale | 188 | 0.01820 | 73 | 40.11 |
| Roxbury | 188 | 0.01511 | 37 | 20.67 |
| Seaport | 192 | 0.04554 | 40 | 22.47 |
| South Boston | 191 | 0.01150 | 45 | 24.86 |
| South End | 188 | 0.01070 | 57 | 32.02 |
| West Roxbury | 189 | 0.01407 | 59 | 32.24 |
|  |  |  |  |  |
| **Total** | **5481** |  | **1370** | **26.88%** |

^1^ Response rates computed using AAPOR Method 3.

**III. Data Collection Results**

The final sample included 1370 completed surveys (1208 paper, 162 online; 30 were completed in Spanish). The number of completed surveys ranged from 37 in Roxbury to 93 in East Boston-Eagle Hill. Overall response rate was 26.88% and ranged from a low of 17.10% in Hyde Park to a high of 40.11% in Roslindale. Full details on each neighborhood sample are presented in Table B1. An additional 256 completed surveys were obtained from members of the previously-constructed Beacon panel, bringing the total number of completed surveys to 1626. Demographics of the sample are reported in Table B2.

| **Race** | | **Income** | |
| --- | --- | --- | --- |
| Asian | 136 (9%) | <$30,000 | 302 (20%) |
| Black | 186 (12%) | $30,000-$49,999 | 196 (13%) |
| Latinx | 164 (11%) | $50,000-$99,999 | 403 (27%) |
| Other | 65 (4%) | $100,000-$150,000 | 243 (16%) |
| White | 1,008 (65%) | >$150,000 | 350 (24%) |

**IV. Weighting of survey data**

The sample requires weighting to account for both differing probabilities of selection and response rates across neighborhoods, especially insofar as these differences create a sample that is demographically and geographically non-representative. We created two survey weights, one for sample design factors including probability of selection and number of adults in the household adjusted for nonresponse bias across neighborhoods, the other which adds a post-stratified weight to account for demographic non-representativeness. Additionally, we conducted this process twice. First, we did it only for respondents to the NSF-Beacon survey. Second, we replicated the procedures for the dataset that combined the NSF-Beacon survey responses with respondents from the previously-constructed Beacon panel (values reported in Table B3 for weighting are highly similar for the NSF-Beacon responses alone and the merged data set).

*Weights for Nonresponse Bias*

Weighting for nonresponse began by neighborhood with the inverse of the probabilities of selection adjusted for the response rates displayed by neighborhood according to the equation (see Table B1 for values):

W_b_ = (Inverse of probability of selection) / (neighborhood response rate)

The final nonresponse adjusted weight further multiplies the base weight by the number of adults 18+ in the household (capped at 4 to prevent excessively large weights). Finally, these weights are adjusted so that the percentage of the total 18+ population in Boston that belongs in each neighborhood agreed with control percentages computed from the 2014-2018 5-year American Community Survey (ACS) data from the Census Bureau. These weights sum to the ACS estimate of the total 18+ population in the city of Boston. Therefore, the final nonresponse adjusted weight can be defined as:

W_NR_ = (W_b_)(number of adults in household)(ACS population adjustment factor)

*Post-Stratified Weights*

As shown in Table B3, even after nonresponse weights, the respondents to the survey were not demographically representative of Boston’s population. Most notably, people with education beyond 4-year college degrees were overrepresented and those with a high school education or less were underrepresented. Women were also overrepresented relative to men and White non-Hispanics were overrepresented relative to Blacks and Hispanics. There was also a smaller age bias with too many 65+ people and too few 18-34. A final adjustment to the survey weights was implemented to adjust for differential survey nonresponse by age, gender, race/Hispanic origin, and education. Control percentages for these categories were computed from the 2014-2018 5-year ACS data. Post-stratification factors were then computed to match weighted survey data to citywide percentages. The final post-stratified weight can be expressed as:

W_PS_ = (W_NR_)(post-stratified factors)

It should be noted, though, that a small amount of trimming of weights, less than one percent of all sample cases, was employed to prevent some extreme values in the post-stratified weights. As shown in Table B3, this additional adjustment process brought the weighted survey estimates much more in line with ACS citywide estimates.

**Table B3. Comparison of ACS controls to nonresponse and post-stratified weights**

|  | **ACS** | **Nonresponse** | | | **Post-stratified** | |
| --- | --- | --- | --- | --- | --- | --- |
| **Age** |  |  | | |  | |
| 18-34 | 46.90% | 38.40% | | | 46.20% | |
| 35-49 | 21.3 | 20.1 | | | 21.5 | |
| 50-64 | 18.4 | 22.1 | | | 18.6 | |
| 65+ | 13.4 | 19.4 | | | 13.7 | |
|  |  |  | | |  | |
| **Gender** |  |  | | |  | |
| Male | 47.60% | 38.00% | | | 47.60% | |
| Female | 52.4 | 62 | | | 52.4 | |
|  |  |  | | |  | |
| **Education** |  | | |  | |  |
| High School including GED or less | 33.60% | 16.40% | | | 32.50% | |
| Some college including 2-year degree | 17.8 | 14.8 | | | 18 | |
| 4-year college degree | 26.5 | 29.3 | | | 27 | |
| Beyond 4-year college degree | 22.1 | 39.5 | | | 22.5 | |
|  |  |  | | |  | |
| **Race/Hispanic origin** |  | |  |  |  |  |
| White non-Hispanic | 49.40% | 57.50% | | | 49.40% | |
| Black non-Hispanic | 20.6 | 15.8 | | | 20.6 | |
| Hispanic | 16.9 | 12.4 | | | 16.9 | |
| Other | 13.1 | 14.3 | | | 13.1 | |

*Appendix C: Full Model Results*

**Table C1.** Parameter estimates from regression models testing the relationship between race, socioeconomic status, and aspects of urban form and patterns of grocery store visits in April 2020, including all control variables.

|  | *Total Visits during COVID* | | *% Visits to $$ Stores* | *% Visits to $ Stores* |
| --- | --- | --- | --- | --- |
|  | Count Model | Zero-Inflation Model |  |  |
| *Same measure Feb. 2020* | 0.01*** (0.0001) | -0.03** (0.01) | 0.42*** (0.02) | 0.40***  (0.02) |
| *Total pop.* | 0.05***  (0.004) | -0.27  (0.23) | — | — |
| *Population density* | -0.11*** (0.007) | 0.20 (0.11) | -1.57*  (0.74) | -0.84  (0.58) |
| *Nearby stores*^a^ | -0.003 (0.006) | 0.25 (0.24) | — | — |
| *Nearby $$ stores*^a^ | — | — | -0.73  (0.79) | — |
| *Nearby $ stores*^a^ | — | — | — | 1.24  (0.67) |
| *% <18 yrs.* | 0.03*** (0.004) | -0.69*** (0.20) | 1.07  (0.63) | -0.19  (0.50) |
| *% >65 yrs.* | -0.01* (0.004) | -0.14 (0.15) | 1.03  (0.57) | -0.27  (0.44) |
| *% Black* | 0.045*** (0.004) | -0.43 (0.38) | 2.73***  (0.59) | -1.02*  (0.47) |
| *% Asian* | -0.007 (0.004) | -0.04 (0.15) | -0.45  (0.54) | 1.05*  (0.42) |
| *% Latinx* | 0.050*** (0.005) | -0.38 (0.33) | -1.16  (0.65) | 2.55***  (0.51) |
| *Med. HH income* | -0.12*** (0.005) | 0.49** (0.18) | 1.49*  (0.64) | -2.23*** (0.51) |
| *% commuting by car* | 0.12*** (0.006) | -0.33 (0.23) | 1.11  (0.82) | 2.37***  (0.67) |
| ***R^2^*** | **.50** | | **.17** | **.20** |

* - *p* < .05, ** - *p* < .01, *** - *p* < .001

*Note:* Sample of 2,327 census block groups (CBG) in metro Boston, MA with population >200 residents and values on all variables. All variables scaled before analysis, meaning parameters are an estimate of the increase or decrease with each change in 1 SD in the predictor variable.

^a^ – Defined as being within a 15-minue drive of the centroid of the CBG.

**Table C2.** Parameter estimates from regression models testing the relationship between race, socioeconomic status, aspects of urban form, and infection rates and patterns of grocery store visits in April 2020, including all control variables.

|  | *Total Visits during COVID* | | *% Visits to $$ Stores* | *% Visits to $ Stores* |
| --- | --- | --- | --- | --- |
| *Same measure Feb. 2020* | 0.01*** (0.0001) | -0.03** (0.01) | 0.44*** (0.02) | 0.39*** (0.02) |
| *Total pop.* | 0.05***  (0.004) | -0.12  (0.25) | — | — |
| *Population density* | -0.11*** (0.008) | 0.18 (0.12) | -2.25**  (0.73) | -0.94 (0.58) |
| *Nearby stores*^a^ | -0.004 (0.006) | 0.26 (0.26) | — | — |
| *Nearby $$ stores*^a^ | — | — | -0.09  (0.79) | — |
| *Nearby $ stores*^a^ | — | — | — | 1.30 (0.67) |
| *% <18 yrs.* | 0.03*** (0.005) | -0.68** (0.23) | 0.72  (0.63) | -0.001 (0.49) |
| *% >65 yrs.* | -0.007 (0.004) | -0.21 (0.17) | 0.93  (0.56) | 0.004 (0.44) |
| *% Black* | 0.050*** (0.004) | -0.22 (0.38) | 2.34***  (0.63) | -1.09*  (0.50) |
| *% Asian* | -0.007 (0.004) | 0.0005 (0.16) | -0.69  (0.53) | 1.18** (0.42) |
| *% Latinx* | 0.050*** (0.005) | -0.48 (0.38) | -1.30*  (0.65) | 2.88***  (0.51) |
| *Med. HH income* | -0.11*** (0.005) | 0.46* (0.20) | 1.39*  (0.63) | -2.16*** (0.50) |
| *% commuting by car* | 0.11*** (0.007) | -0.34 (0.26) | 0.71  (0.84) | 2.56***  (0.68) |
| *Infection rates (April 2020)* | -0.005** (0.002) | -0.07 (0.08) | — | -0.02  (0.15) |
| ***R^2^*** | **.60** | | **.19** | **.22** |

* - *p* < .05, ** - *p* < .01, *** - *p* < .001

*Note:* Sample of 2,327 census block groups (CBG) in metro Boston, MA with population >200 residents and values on all variables. All variables scaled before analysis, meaning parameters are an estimate of the increase or decrease with each change in 1 SD in the predictor variable.

^a^ – Defined as being within a 15-minute drive of the centroid of the CBG.

**Table C3.** Parameter estimates from regression models testing the relationship between race, socioeconomic status, and aspects of urban form and patterns of grocery store visits in Boston only in April 2020, including all control variables.

|  | *Total Visits during COVID* | *% Visits to $$ Stores* | *% Visits to $ Stores* |
| --- | --- | --- | --- |
| *Same measure Feb. 2020* | 0.01*** (0.0003) | 0.28*** (0.06) | 0.20***  (0.05) |
| *Total pop.* | 0.13***  (0.01) | — | — |
| *Population density* | -0.12*** (0.01) | -2.78*  (1.13) | -0.05  (0.79) |
| *Nearby stores*^a^ | -0.05**  (0.02) | — | — |
| *Nearby $$ stores*^a^ | — | -8.00**  (3.00) | — |
| *Nearby $ stores*^a^ | — | — | -0.28  (2.15) |
| *% <18 yrs.* | 0.06***  (0.01) | 0.82  (1.69) | 0.38  (1.17) |
| *% >65 yrs.* | -0.01  (0.01) | 1.01  (1.51) | 0.62  (1.04) |
| *% Black* | 0.14***  (0.01) | 1.32  (1.22) | -0.02  (0.85) |
| *% Asian* | 0.04**  (0.01) | -0.98  (1.49) | 2.30*  (1.04) |
| *% Latinx* | 0.17***  (0.01) | -1.71  (1.65) | 4.38***  (1.15) |
| *Med. HH income* | -0.05*  (0.02) | -0.07  (2.30) | 0.16  (1.61) |
| *% commuting by car* | 0.06***  (0.02) | -0.64  (2.39) | 1.21  (1.72) |
| ***R^2^*** | **0.66** | **.10** | **.17** |

* - *p* < .05, ** - *p* < .01, *** - *p* < .001

*Note:* Sample of 531 census block groups (CBG) in Boston, MA with population >200 residents and values on all variables. All variables scaled before analysis, meaning parameters are an estimate of the increase or decrease with each change in 1 SD in the predictor variable.

^a^ – Defined as being within a 15-minute drive of the centroid of the CBG.

**Table C4.** Parameter estimates from regression models testing the relationship between race, socioeconomic status, aspects of urban form, infection rates, and perception of risk and patterns of grocery store visits in Boston only in April 2020, including all control variables.

|  | *Total Visits during COVID* | *% Visits to $$ Stores* | *% Visits to $ Stores* |
| --- | --- | --- | --- |
| *Same measure Feb. 2020* | 0.01*** (0.0003) | 0.28*** (0.06) | 0.20***  (0.05) |
| *Total pop.* | 0.14***  (0.01) | — | — |
| *Population density* | -0.13*** (0.01) | -2.76*  (1.14) | 0.05  (0.80) |
| *Nearby stores*^a^ | -0.04*  (0.02) | — | — |
| *Nearby $$ stores*^a^ | — | -8.58**  (3.07) | — |
| *Nearby $ stores*^a^ | — | — | 0.27  (2.18) |
| *% <18 yrs.* | 0.06***  (0.01) | 0.90  (1.70) | 0.45  (1.18) |
| *% >65 yrs.* | -0.02  (0.01) | 1.17  (1.53) | 0.38  (1.05) |
| *% Black* | 0.14***  (0.01) | 1.11  (1.29) | -0.23  (0.90) |
| *% Asian* | 0.03**  (0.01) | -0.78  (1.50) | 2.13*  (1.05) |
| *% Latinx* | 0.17***  (0.01) | -1.76  (1.69) | 4.10***  (1.19) |
| *Med. HH income* | -0.05**  (0.02) | 0.16  (2.32) | -0.07  (1.62) |
| *% commuting by car* | 0.06***  (0.02) | -0.78  (2.40) | 1.13  (1.72) |
| *Perceived infection risk* | -0.10**  (0.03) | 5.62  (4.82) | -2.09  (3.37) |
| *Infection counts (April 2020)* | 0.003  (0.002) | -0.14  (0.33) | 0.32  (0.23) |
| ***R^2^*** | **.66** | **.11** | **.17** |

* - *p* < .05, ** - *p* < .01, *** - *p* < .001

*Note:* Sample of 531 census block groups (CBG) in Boston, MA with population >200 residents and values on all variables. All variables scaled before analysis, meaning parameters are an estimate of the increase or decrease with each change in 1 SD in the predictor variable.

^a^ – Defined as being within a 15-minute drive of the centroid of the CBG.

**Table C5.** Parameter estimates from regression models testing the relationship between race and socioeconomic status and number of residents working in delivery and the effect of deliveries on total visits to grocery stores.

|  | *# Residents Working in Delivery*^a^ | *Total Visits during COVID* | |
| --- | --- | --- | --- |
|  |  | Count Model | Zero-Inflation Model |
| *Same measure Feb. 2020* | — | 0.01*** (0.0001) | -0.03** (0.01) |
| *Residents in Delivery* | — | 0.001***  (0.0001) | -0.10*  (0.05) |
| *Total pop.* | 1.94***  (0.14) | 0.05***  (0.004) | -0.5  (0.23) |
| *Population density* | -0.84***  (0.20) | -0.11*** (0.007) | 0.21 (0.11) |
| *Nearby stores*^b^ | — | -0.003 (0.006) | 0.26 (0.25) |
| *% <18 yrs.* | -0.48**  (0.17) | 0.03*** (0.005) | -0.68*** (0.20) |
| *% >65 yrs.* | -0.63***  (0.16) | -0.01* (0.004) | -0.18 (0.15) |
| *% Black* | 0.90***  (0.16) | 0.044*** (0.004) | -0.50 (0.40) |
| *% Asian* | -0.14  (0.15) | -0.005 (0.004) | -0.04 (0.16) |
| *% Latinx* | 1.27***  (0.18) | 0.048*** (0.005) | -0.36 (0.34) |
| *Med. HH income* | -1.37***  (0.18) | -0.11*** (0.005) | 0.47*  (0.18) |
| *% commuting by car* | 1.28***  (0.21) | 0.11*** (0.006) | -0.28 (0.23) |
| *R^2^* | **0.20** | **0.60** | |

* - *p* < .05, ** - *p* < .01, *** - *p* < .001

*Note:* Sample of 2,351 census block groups (CBG) in metro Boston, MA with population >200 residents and values on all variables. All variables scaled before analysis, meaning parameters are an estimate of the increase or decrease with each change in 1 SD in the predictor variable.

^a^ – As inferred by SafeGraph as the number of devices that stopped for <20 minutes at >3 locations outside of their home area.

^b^ – Defined as being within a 15-minute drive of the centroid of the CBG.

**Table C6.** Parameter estimates from regression models testing the relationship between race and socioeconomic status and exposure to infection from grocery store visits in April 2020 taking into account strategies for diminishing exposure, including all control variables.

|  | *Total Exposure through Visits* | *Avg. Exposure per Visit* | *Avg. Exposure per Visit* | *Avg. Exposure per Visit* |
| --- | --- | --- | --- | --- |
| *% visits to $ stores* | - | - | 0.13***  (0.004) | - |
| *% visits to $$ stores* | - | - | - | -0.18***  (0.004) |
| *Total Pop.* | 0.22***  (0.006) | -0.06***  (0.005) | -0.06***  (0.005) | -0.06***  (0.005) |
| *Population density* | -0.02*  (0.009) | 0.10***  (0.004) | 0.10***  (0.004) | 0.08***  (0.004) |
| *% <18 yrs.* | 0.028***  (0.02) | -0.03***  (0.004) | -0.03***  (0.005) | -0.04***  (0.005) |
| *% >65 yrs.* | -0.01  (0.008) | -0.04***  (0.005) | -0.04***  (0.005) | -0.04***  (0.005) |
| *% Black* | 0.33***  (0.006) | 0.27***  (0.004) | 0.28***  (0.004) | 0.30***  (0.004) |
| *% Asian* | 0.15**  (0.007) | 0.15***  (0.004) | 0.14***  (0.004) | 0.14***  (0.004) |
| *% Latinx* | 0.27***  (0.007) | 0.21***  (0.005) | 0.18***  (0.005) | 0.19***  (0.005) |
| *Med. HH income* | -0.28***  (0.01) | -0.06***  (0.006) | -0.04***  (0.006) | -0.04***  (0.006) |
| *% commuting by car* | -0.04***  (0.01) | -0.12***  (0.006) | -0.14***  (0.006) | -0.13***  (0.006) |
| *Infection rates (April 2020)* | 0.10*** (0.006) | 0.13*** (0.003) | 0.13*** (0.004) | 0.13*** (0.003) |
| *R^2^* | **.39** | **.38** | **.39** | **.40** |

* - *p* < .05, ** - *p* < .01, *** - *p* < .001

*Note:* Sample of 2,153 CBGs in metro Boston, MA with population >250 residents, at least one recorded grocery store visit in April 2020, and values on all other variables. The outcome variables were modeled as a Poisson distribution. All predictor variables scaled before analysis, meaning parameters are an estimate of the likelihood of increase or decrease with each change in 1 SD in the predictor variable. All models also controlled for the same measure in Feb. 2020, total population, population density, total stores or $$ or $ stores within a 15-minute drive of the centroid of the CBG (as appropriate per model), % < 18 yrs., % > 65 yrs., and % commuting by car. See Methods for variable details.
